# Sex and gender influence on immunity and autoimmunity

**DOI:** 10.3389/fimmu.2023.1142723

**Published:** 2023-05-26

**Authors:** Robert G. Lahita

**Affiliations:** ^1^ Hackensack Meridian School of Medicine, Hackensack, NJ, United States; ^2^ Institute for Autoimmune and Rheumatic Disease, St. Joseph Health, Wayne, NJ, United States

**Keywords:** prolactin, DHEA, SLE - systemic lupus erthematosus, testosterone (androgen), estrogen, genetic, epigenetics, progesterone

## Abstract

Autoimmune diseases are skewed toward one biological sex or another. This is the obvious observation of many decades, and it remains unexplained. Females predominate with most autoimmune diseases. The reasons for this predilection are an interplay of genetic, epigenetic and hormonal factors.

## Introduction

The predilection of certain diseases for biological sex is well known and some of the biological reasons for this sexual skew are becoming clear. Clinical observation of patients with autoimmunity over many years and during infections like Covid19 indicate that biological sex is important to disease progress and often the eventual outcome. In this brief review both basic and clinical observations help us understand how biological sex affects immune disease.

In the case of autoimmune disease, early animal data and clinical observation indicated that sex steroids like estrogen and testosterone played an important part in the clinical manifestations of disease (1). The disease systemic lupus erythematosus (SLE) is one of those clinical conditions where studies have been extensive (2).

Autoimmune diseases are likely caused by an array of factors: genetic predisposition, epigenetic regulation (miRNA, DNA methylation, and histone modification), infections resulting in antigenic mimicry, or some other reasons for activation of the immune system.

**Table 1** shows the known sex predilection of some autoimmune diseases in the human.

**Table 1:** Sex ratios of autoimmune diseases during early adulthood (age 15-40 years).

Disease	Female percentage	Male percentage
Erythema nodosum	90	10
Systemic Lupus Erythematosus	90	10
Takayasu Arteritis	85	15
Ulcerative Colitis	75	25
Thrombocytopenic purpura	75	25
Erythema multiforme	65	35
IgA Nephropathy	35	65
Goodpasture Syndrome	15	85

Adapted from Medical Hypotheses Volume 151, June2021.

## Genomic pathways of action

Estrogen receptors are part of classical genomes (3). Estradiol binds to its cognate intracellular steroid receptor (either ER alpha or ER beta). Estrogens bind to the cytoplasmic receptor causing conformational change which through a series of processes go into the nucleus to cause gene expression (4). These receptors are largely in reproductive tissue, but also in the immune system. They affect both innate and adaptive immunity. Activated T cells have estrogen receptors and both mRNA and protein levels of estrogen receptors have been described for T cells, B cells, monocytes, and dendritic cells (5) (6). High levels are found in CD4 T cells, whereas CD8 T cells and monocytes express low levels of both receptors. Non genomic pathways of action involve crosstalk with signaling.

## Micro RNA

MicroRNA (miRNA) is very important to post translational gene regulation This is a powerful mechanism of gene regulation in health and disease. Though the miRNA is roughly 3% of the human genome, there is a possibility that over 30% of human genes might be regulated by miRNA (7). MiRNA are short 22-nucleotide non-coding RNAs transcribed from genomic DNA. These block translation or lead to degradation of miRNA. Many miRNAs are coded on the X chromosome. X linked miRNAs likely contribute to sex bias in autoimmunity (8, 9). Several immunosuppressive genes are targeted by X-linked miRNA (fork head box P3, CTLA4, Casitas B lineage lymphoma, CBL-B suppressors of cytokine signaling genes, and apoptosis. Estrogens upregulate many miRNAs, but data from murine models also show that miRNA can be decreased in response to estrogen.

It is extraordinary that the majority of sex differential expression of miRNAs occur in the brain. There may also be an interplay between gonadotrophin releasing hormone and miRNA.

## Genetics and epigenetics

Numerous genes are in part regulated by sex hormones. This happens through G protein signaling and membrane regulation. Genetic and epigenetic factors are responsible for the sexual dimorphism of the immune system (10). These factors influence the development of autoimmune disease (11–13).

HLA genes on chromosome 6 include the HLA DQ and DR genes. These play a central role in the presentation of antigens to CD4+ T cells. Females have a higher frequency of HLA D4 than men. In diseases like SLE or RA there is a greater risk of disease for women.

Supporting non hormonal factors in the onset of diseases like SLE, there are minimal effects of hormones before and after puberty when sex hormone levels are minimal. In the case of the autoimmune disease SLE, prepubertal girls and postmenopausal women develop the disease at a higher rate than age matched males. In addition, there is no evidence that hormonal replacement therapy for women increases the appearance of disease (14).

The genes on both the X and the Y chromosome play a significant role in the incidence of immune disease. The X chromosome has 1100 genes, and the Y chromosome has 100 genes. Gene expression for autoimmune disease in both males and females is on the X chromosome.

X inactivation during embryogenesis is critical to gene expression in females. Autoimmune phenomena are observed in X linked primary immunodeficiencies. Moreover, the X chromosome plays a major role in autoimmunity (15). Skewed X chromosome inactivation is found in many autoimmune diseases. There is a methylome and transcriptome that have been identified as sex specific (2, 16).

Klinefelter males have an extra X chromosome (XXY) and are 14-fold more likely to get an autoimmune disease like SLE (17) or RA. Moreover, there are other conditions that occur in Klinefelter males such as chronic liver disease and in at least one case porphyria as well, all of them estrogen dependent diseases. Case reports of Turner syndrome associated autoimmune diseases also exist. Turner syndrome patients are devoid of two X or one Y chromosome and have only one X (45 chromosomes) (18).

The X chromosome encodes several immune associated genes: CD40L, CXCR3, OGT, FOXP3, TLR7, TLR8, IL2RG, BTK and IL9R (19). In addition to protein coding genes, microRNAs encoded on sex chromosomes may contribute sex differences in SLE, because these (20) are enriched on the X chromosome with some 7% of microRNAs encoded there. The real function of these RNAs is not fully known, but it is apparent that 18 of the linked miRNAs are expressed on the X chromosome, whereas none are expressed on the Y chromosome (8, 21).

Despite having 2X chromosomes in females, one of the X chromosomes is silenced through X inactivation (22). X inactivation is initiated by transcription of the long non-coding RNA (Xist from the X that will become inactive. This happens through epigenetic processes.

About 15% of genes on X consistently escape inactivation, whereas 10% variably escape X inactivation. Expression of these genes from the Xi relative to the X ranges from a few percent to equivalent expression and results in female biased expression that may contribute to sexual dimorphism. In diseases like SLE, T cells exhibit abnormal upregulation of X-linked genes associated with mis localization of Xist RNA (23). Demethylation of regulatory sequences on Xi results in upregulation of CD40L expression in SLE. All of this means that epigenetic dysregulation of the X chromosome might promote sex bias in diseases, like SLE (24, 25).

In SLE there are also SNPs on autosomes that are associated with sex skewing of disease. This is outside of sex chromatin. Gene/sex interactions has been demonstrated for select autoimmune susceptibility loci including FCER1A, Osteopontin, HLA regions 1 and 2, IRF5, and KIAA1542 (26).

Steroids enter cells and upon binding to their receptors, engage with specific DNA elements and steroid responsive elements, to regulate transcription of target genes (27). Hormonal and genetic/epigenetic factors interact to influence sex predilection of autoimmune diseases like SLE. Estrogen alone regulates expression of genes like IFN gamma, IRF5, and TLR8 potentiating female expression. No doubt hormones like estrogen and testosterone are epigenetic regulators. In T cells estrogen reduces DNMT1 expression *via* miR148a, leading to global hypomethylation (28).

## Hormone effects

### Estrogens

As mentioned above, hormones have a major role in both the pathogenesis and expression of autoimmune disease and they have a major role in the etiology, since many of the signs and symptoms of autoimmunity occur before puberty and after menopause when hormones are least active (3, 29). Older studies showed that inbred strains of mice prone to getting SLE (NZB/W F1) could have their disease ameliorated with ovariectomy or injection of androgens showing the importance of estrogens (1). More recently animal models were revealing. SNF1 female mice develop spontaneous immune complex mediated glomerulonephritis (GN) and treatment of male mice with 17β estradiol increased their mortality. Treatment of various strains of mice like the MRL/lpr strain or the NZB/W strains with estrogens or ERα agonists increase murine mortality and immune complex GN. Recent experiments to explain mechanisms have been quite clear. ERα knock-out mice (KO) eliminates lupus -like autoimmune disease in specific lupus prone strains. Plasmacytoid dendritic cells from ERα-/- NZM2410 mice have a reduced type 1 interferon signature independent of plasmacytoid dendritic cells.

Human studies have evaluated the role of estrogen blockade in human disease. Experiments from a placebo controlled double blind trial of patients randomized to receive fulvestrant (an ERα antagonist) or placebo showed no real clinical difference in serologic manifestation of SLE (30). During the human study, gene expression and signaling pathway analysis revealed significant changes in T helper cell differentiation, steroid receptor signaling, protein ubiquitination, and sumoylation pathways indicating that the altered estrogen receptor and glucocorticoid receptor contributed to SLE sexual dimorphism.

There were many early attempts to use estrogen antagonists like Tamoxifen in the treatment of the disease lupus. None were successful (31).

Estrogens play a major role in regulation of the immune system and disease (32). Studies have shown that miRNA expression in both lymphoid and non-lymphoid cells is regulated by estrogen. Estrogen expression and estradiol regulated mRNA (protein coding genes) contribute to estrogen regulation of proinflammatory and autoimmune events. Estradiol confers protection against HIV and other sexually transmitted diseases through enhancement of CD4 T cells (33). Estrogen exaggerates systemic lupus by upregulating TH2 cytokines (Il-4) that activate B cells and allow the release of autoreactive B cells (3). Estrogen also increases antibody against conserved molecules like DNA and the hormone enhances immune reactivity against autoantigens like phospholipids. Finally, estrogen acts on increasing inflammation and expression of B cell activating factor (BAFF and Blys) and interferon signature genes.

Not surprisingly, Blys and interferon signature suppression have become the newest treatments for systemic lupus.

Complexity arises when one considers the many metabolites of estrogen available to receptors and their effects on miRNA. Some estrogens have greater affinity than others regarding the estrogen receptor (34–36). The varying female hormonal milieu across the estrus cycle, and the fact that estrogen peaks during the ovulatory phases in mammals, means that there can be both biological and clinical differences observed in human disease during these times.

In human peripheral blood, mononuclear cells (PBMCs) were stimulated by levels of estrogen *in vitro*. Estrogen triggered TNF-alpha and Il-6 production in male but no female derived PBMC.

Estrogen is implicated in neutrophil apoptosis, chemotaxis, and the formation of extracellular NETs or extracellular chromatin fibers capable of binding pathogens. Female derived neutrophils have reduced apoptosis when compared to males (37).

### Prolactin

Immune cells, including lymphocytes, secrete prolactin (38, 39). There is abundant data in humans that hyper-prolactinemia is associated with an increase of disease activity (40, 41). This is certainly the case in human lupus. Prolactin is elevated in women during pregnancy and breastfeeding. Hyperprolactinemia can be found in men and women with microadenomas of the pituitary (42). The presence of hyperprolactinemia in SLE prompted studies of the effect of bromocriptine in the treatment of lupus. According to the clinical studies of seven patients, their SLAM and SLEDAI scores (two measures of clinical activity in this disease) and the level of anti-DNA antibodies decreased. A larger study (placebo controlled) of 36 bromocriptine and 30 placebo patients showed that bromocriptine improved SLEDAI scores significantly by the fifth visit and that there were less clinical flares among those on the drug. To evaluate prolactin in a post-partum group of women, a study of 76 pregnant SLE patients, 38 of whom received bromocriptine right after delivery. In the control group 14 patients had a flare of disease, whereas only 6 patients in the bromocriptine treated group had a flare. The bromocriptine group also had lower use of corticosteroids (40, 43).

From murine studies, B cell tolerance breakdown occurs in those animals treated with estrogen (44). Prolactin increases estrogen which could explain the sexual dimorphism in mice. BALB/c mice treated with both bromocriptine and estrogen had reduced anti-DNA antibodies as well as less glomerulonephritis. The theory is that prolactin promotes the survival of autoreactive clones of B cells (45, 46).

Prolactin receptors are found on monocytes and T lymphocytes. The way prolactin works on immunity is through T lymphocytes. Prolactin mediates T-bet, a transcription factor involved in the production of Th-1type cytokines, including interferons (47). This is the way this hormone regulates T cell mediated inflammatory reactions. Elevated levels of prolactin can be found in the spinal fluid of patients with neuropsychiatric lupus (42). Thyroid antibodies are also raised in those patients with hyper-prolactinemia (48).

### Progesterone

Progesterone has an overall immunosuppressive effect on innate immune cells. Progesterone suppresses NET formation and NETosis and can diminish the effects of estrogen on NET formation. Like estrogen this depends on progesterone concentration. Not surprisingly, progesterone has also been studied in lupus patients, mainly because the levels of progesterone in female patients with active lupus are quite low in the luteal phase, suggesting an inverse correlation of levels with lupus activity. In fact, the use of low dose progesterone-only oral contraceptives is often said to be a good for patients with autoimmune disease. A randomized placebo-controlled trial of a progesterone -only hormone replacement did not exacerbate autoimmune diseases. Clinical studies support a protective role for progesterone against diseases like SLE, but the overall significance of this hormone’s use in autoimmune disease is still under investigation. The data from animal studies (murine mostly) are contradictory and not clear (49, 50).

### Androgens

Androgens have a unique place in the study of the immune system in disease since they are the precursors of all estrogens. Early work with androgen metabolism in lupus patients indicated that women had very low levels of androgens like testosterone and DHEA in blood, prompting several studies of use of androgen to treat patients (51). Testicular hypofunction is often associated with autoimmunity in males adding more importance to this hormonal group. The oxidation of testosterone in SLE women is increased as is androgen oxidation in patients with Klinefelter syndrome (XXY).

Androgen use as chemotherapy has been shown to suppress immunoglobulins, in particular IgA (52, 53).

Early studies in mouse lupus models showed that androgens ameliorated lupus-like activity. Orchiectomy of male NZB/W mice increased mortality, which could be reversed with dihydrotestosterone treatment (54). Ovariectomized female mice given androgens had better survival than mice treated with estrogen. These murine studies formed the basis for the study of androgens as a treatment of lupus in the human (55).

The data from humans also suggested that androgen therapy could be helpful. There were numerous studies of androgens like 19-nor testosterone, and dehydroepiandrosterone (DHEA) in the treatment of human disease (51, 56). In a double randomized placebo-controlled study on severe SLE, 21 patients were studied (57). Nine DHEA patients and 10 placebo patients completed the trial, and although the DHEA patients trended towards stabilized nephritis, hematologic lupus and serositis, there was no significant difference between the groups when the activity measures were studied. (SLEDAI and SLAM) (57). When large numbers of patients were tried on 100 mg or 200 mg per day and compared against the placebo group, the steroid doses could be lowered, and the overall flare rate decreased. Larger studies involving as many as 381 lupus patients with SLE that were randomized against placebo showed no difference in overall clinical outcome (56, 58, 59).

Studies of DHEA have continued and there is evidence that patients with severe disease might improve on this drug, but overall, the data are unclear regarding the use of male hormones to treat SLE or any other autoimmune disease (60).

There are suggestions from recent data that DHEA might have protective effects. The peripheral blood lymphocytes of 20 SLE untreated patient with sex and age matched controls revealed that the lymphocytes from the DHEA treated patients had lowered rates of apoptosis and increased mRNA expression of BCL-2, providing another mechanism by which DHEAS (sulfated form) might prevent autoimmunity (61).

## Summary

Sex and gender have a significant effect on the immune system. The interplay between genetics and sex steroid metabolism within the body is how the immune response handles infections and regulates immune function. The reasons for the female predilection for autoimmune disease remains unknown but is likely due to the genetics of the X chromosome, hormonal regulation of immune cells (CD4 T cells, monocytes, B cells and others) and the epigenetics affected by sex hormones. The clinical extent and severity of autoimmune disease depends on the complex interplay of genetics and hormonal metabolism, factors like miRNA, interferon signaling, and B lymphocyte stimulating factor (62).

## Author contributions

The author confirms being the sole contributor of this work and has approved it for publication.
